# Contaminants in human nail dust: an occupational hazard in podiatry?

**DOI:** 10.1186/1757-1146-7-15

**Published:** 2014-02-20

**Authors:** Paul D Tinley, Karen Eddy, Peter Collier

**Affiliations:** 1 Podiatry Department, School of Community Health, Charles Sturt University, PO Box 789, Albury, NSW 2640, Australia

## Abstract

**Background:**

There has been limited literature indicating that podiatrists’ health may be at risk from exposure to human nail dust. Previous studies carried out in the UK have shown that large amounts of dust become airborne during the human nail drilling procedure and are present in the air up to 10 hours after a clinical session. This increases the risk of Respiratory Tract (RT) infection for the practitioner.

**Methods:**

This study used a nasal swabbing technique and fungal culture to determine whether podiatrists (n = 50) had the same microbes present in their nasal cavities as non-podiatry health professional control group (n = 45). All swabs were cultured, counted and identified for each subject. Survey data of use and type of nail drill, type of mask used and frequency of change over a two week period.

**Results:**

The results showed podiatrists had a greater range of microbes in their nasal cavities although the controls had greater overall numbers of organisms. The known pathogen and common mould, Aspergillus fumigatus was ost commonly found fungus within the podiatric group with 44% of the group having the fungus present. All nail drills used by the podiatrists had some form of dust extraction (except one). Of concern was 17% (n = 8) of the podiatrists did not use a mask at all whilst drilling and seemed unaware of any infection control issues. Simple disposable masks were the most frequently worn with only half being changed after each patient further increasing the cross infection risk

**Conclusion:**

The high levels of Aspergilus contamination is a significant finding in the podiatry group as this fungus is small enough to enter the tissue of the nasal cavity and as a small particle will stay airborne in the room for up to 16 hours. Aspergilus has been shown to cause brain and soft tissue tumours in extreme cases. The high levels of upper respiratory track problems reported in the literature may well be caused by this fungal agent. The non use and use of inappropriate masks by podiatrists is clearly an occupational hazard to their health and well being.

## Background

Podiatrists reduce thickened toe-nails using high speed drills as part of their general practice, which creates fine airborne particles of dust that can be inhaled by practitioners. Studies have shown large amounts of nail dust becomes airborne, with the smallest particles still present in the air up to 10 hours after a clinical session [1,2]. Studies have also found an association between asthma and the increasing number of years podiatrists have been in practice. Conjunctivitis, rhinitis and eczema have also been linked with exposure to nail dust [3-5].

Although the National Occupational Health and Safety Commission [6] recognises dust as a hazard in the workplace, there are no specific guidelines in place in Australia for human nail dust. In most countries dust extraction or water sprays systems are now industry standards for podiatrists. The use of personal protective equipment such as masks and gloves during human nail drilling are considered industry standards for occupational health and safety requirements [7-11]. A number of researchers [2,7,9,12] agree that no drill dust extraction system will completely remove all dust particles from the air. In fact, Purkiss [2] found that many of the masks worn were not able to filter the smaller particles with 99% of airborne dust particles being smaller than 5 microns (5 μm) and 70% of those being smaller than 0.8 μm making them very hard to protect against with face masks.

Many different fungal, yeast and bacterial organisms have been cultured from infected toe nails. Some of these microorganisms, such as *Candida*, *Aspergillus*, *Fusarium* and *Staphylococcus aureus* are known to cause serious diseases once they enter the body [13]. Curtis *et al.*[14] suggests that people who are exposed to mould had a wide range of health problems with the most common being fatigue, muscle/joint problems, rhinitis, sinusitis, neurocognitive dysfunction and respiratory problems. Abramson and Wilton [1] analysed the contents of nail dust and found it was made up of live fungal spores and filaments. The most common fungal organism detected in infected nails, the *Trichophyton sp.*, has been implicated in causing allergic reactions in susceptible people [15], with several studies [16-18] finding podiatrists had high levels of antibodies to these organism in their bloodstream. Similar allergens are now accepted as a cause of occupational asthma [19,20].

Professional 'burnout’ has been identified as a major problem in the practice of podiatry [21]. Working with patients and the risk of cross infection has been shown to be one of the major contributors to this 'burnout’ problem. Identifying risk factors in the perception of practitioners working with infectious material is critical for the understanding of strategies that would better the podiatry profession to work in a healthy and safe workplace.

The primary aim of this study was to determine if podiatrists have a different species of microbes present in the nasal cavity compared to a group of subjects that have not been exposed to large amounts of human nail dust particles as part of their work.

## Methods

### Study population

Fifty volunteer podiatrists age 31.7, SD 9 years from a potential sample group of 200 podiatrists attending a rural podiatry conference formed the basis of this study. Forty five occupational therapy students aged 28.0, SD 5 years volunteered to act as the comparison sample group in the study. The occupational therapy students were closely aged-matched with the podiatry sample group compared to other student groups and they had not been involved with clinical placement before the testing occasion. Convenience sampling was used for the recruitment of both sample groups. Written consent was obtained prior to the nasal swabbing technique. This research project was approved by the Charles Sturt University’s Ethics in Human Research Committee. Any participant who admitted to having an upper respiratory tract infection or seasonal allergic rhinitis was excluded from the study.

### Data collection

Each participant was instructed to self administer one nasal swab per nostril by placing a sterile cotton tipped applicator two centimetres into each nasal cavity and rotating the swab against the nasal mucosa. The swabs were then taken to the university’s laboratory and cultured onto a Petri dish containing Sabouraud dextrose agar and a second separate slope containing Sabouraud dextrose agar with the addition of Chloramphenicol, Gentamycin and Actadione to inhibit moulds and bacteria and encourage the growth of dermatophytes. The plates and slopes were incubated at room temperature for 3 weeks and examined weekly for fungal growth. The identification of the fungi was based on growth rate, gross colony appearance and microscopic morphology by the laboratory technician and by use of text books [22,23]. Gram staining was used for the bacterial species with further cultures taking place on blood agar and McConkey’s agar. The fungi were examined by using a transfer medium to view the colony growth on a glass slide with ink in the preparation. All were then examined under a compound light microscope. Six unidentifiable samples including a bacteria isolate were sent to a commercial diagnostic laboratory for identification with three being formally identified.

The podiatrists were also asked to fill out a closed-ended five point questionnaire which identified the number of years they had been in practice, the number of times they had used a nail drill in the previous two weeks, the type of dust extraction mechanism used, the type of facial mask used and how frequently it was changed. The podiatrists ticked a box next to an answer among several choices that best suited their response. As the responses were coded, i.e. each response given a numeric code, and descriptive data collected using frequency counts and percentages for each question. Windows Excel^©^ (Microsoft Corporation, Redmond, WA) was used to compile the results and prepare the graphs.

## Results

A total of 134 colonies of 11 different fungal and yeasts were isolated as outlined in Table 1. Some plates had mixed cultures which were counted as separate colonies.

**Table 1:** Types of fungi cultured from podiatrists and controls

**Type of fungus**	**Podiatrists N = 50**	**%**	**Controls N = 45**	**%**
*Aspergillus fumigatus*	22	44	4	9
*Aspergillus versicolor*	0	0	3	7
*Penicillium spp*	13	26	10	22
Unidentified yeast	3	6	20	44
*Scytilidium spp*	1	2	0	0
Unidentified dematiaceous fungus	17	34	15	33
*Bacillus*	1	2	0	0
*Alternaria*	1	2	0	0
*Phaeoannellomyces werneckii*	1	2	8	17
*Staphylococcus aureus*	1	2	0	0
*Microsporum ferrugineum*	1	2	0	0
*Hyaline hyphomycetes*	1	2	4	9
Unidentified	3	6	7	15

The podiatrists’ results showed 44% having *Aspergillus fumigatus* isolated from their nasal cavities compared to only 9% of the control group. The control group had 7% displaying *Aspergillus versicolor* in their noses whereas this species was not found at all in the podiatrists. 44% of the control group had unidentified yeasts present in their noses compared with only 6% found in the podiatrists. As expected, comparable amounts of the common environmental *Penicillium* and dematiaceous fungi were isolated in both groups. *Phaeoannellomyces werneckii* was found in 17% of the controls but *Alternaria* and *Scytilidium spp*. were only found in the podiatrists. Two different types of bacteria were cultured from the podiatrists. One dermatophyte, *Microsporum ferruginieum* was cultured from the podiatrists and both groups had some moulds that were unable to be identified by either the researchers or the pathology laboratory. The researchers were surprised by the lack of *Trichophyton rubrum* identified in the colonies cultured. *Trichophyton rubrum* is a commonly occurring dermatophyte found in nail tissue. This may have been due to the sensitivity of the slopes used and the difficulties in growing these organisms.

**Figure 1:**
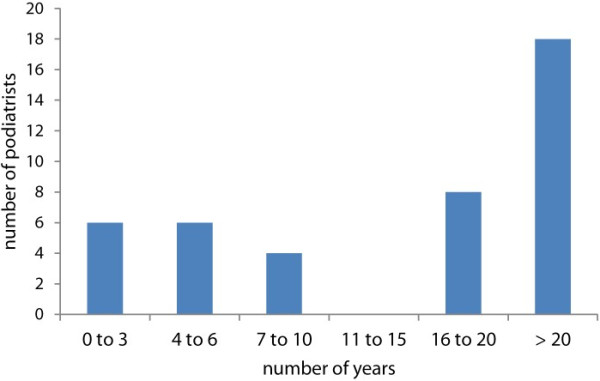
Number of years practicing as a podiatrist.

Although there was a 100% participation rate in questionnaires being completed, some individual questions were not answered. The podiatrists were asked to respond to how many years they had been practising podiatry. The range was between 0 years and over 20 years as depicted in Figure 1. The mean years the group had been practising were 11-15 years. One person did not answer the question.

**Figure 2:**
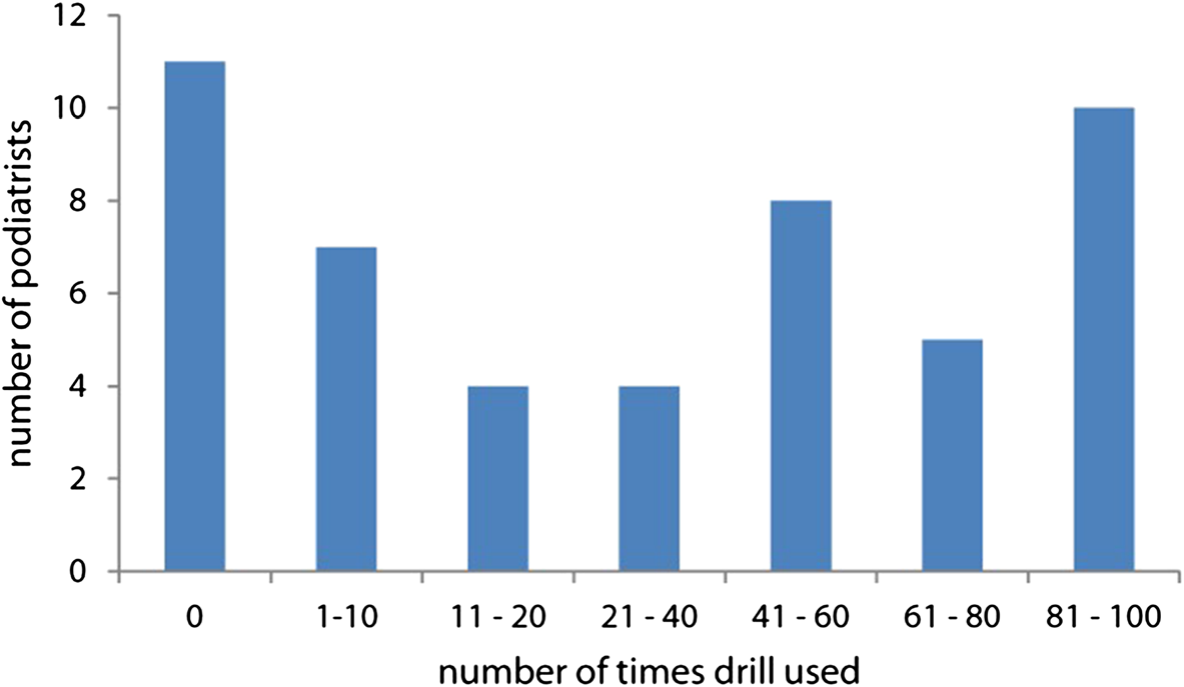
Drill usage in podiatry sample group over previous two week period.

The respondents were questioned as to the number of times they had used a drill in the last two weeks. The results are depicted in Figure 2. Although some podiatrists had answered 'nil times’ they still indicated on further questions that they had a drill so it cannot be ascertained if they were on leave or had not used the nail drill just in the last two weeks.

A review of the raw data showed that the podiatrists who had not used a nail drill in the previous two weeks did not own a nail drill, which was based on the type of response to the question on type of nail drill extraction. Of the 45 podiatrists who responded that their drill had a dust extraction system, most were of the vacuum type. One mentioned that their extra nail drill used on domicilliary visits did not have any dust extraction.

**Figure 3:**
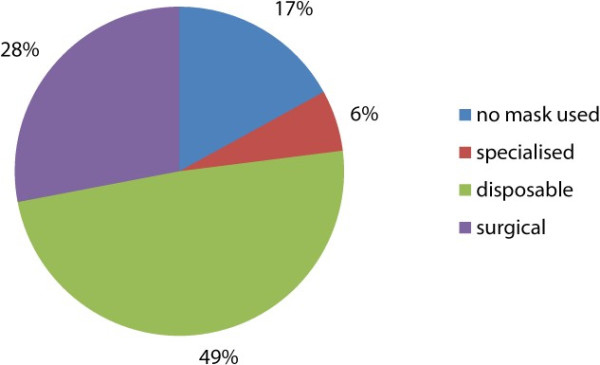
Type of mask worn by podiatrist during nail reduction.

Figure 3 showed that 81% of the podiatrists who had used a drill in the last two weeks had used some form of a mask to prevent inhalation of dust. There were 17% who did not wear a mask at all whilst drilling although this could be higher as some indicated only occasional use of a mask. One respondent claimed they only wore a mask whilst using the nail drill that did not have dust extraction i.e. on domiciliary visits. This podiatrist did not show any significant difference to the other more experienced podiatrists in the sample group.

**Figure 4:**
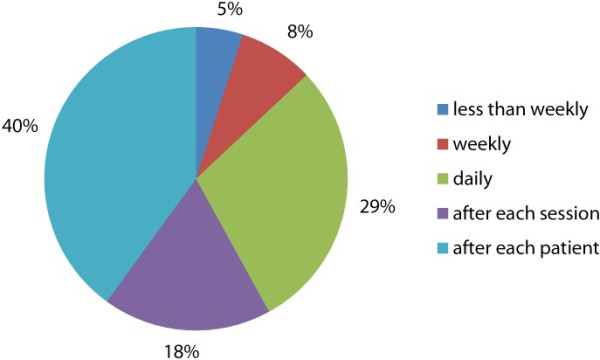
Pie chart of how often mask is changed by podiatrist.

The frequency in which podiatrists changed their masks was of interest Figure 4. Nearly half the respondents claimed they changed the mask after each patient. The two podiatrists who used more specialised masks stated that they changed their masks less frequently which may be recommended by the manufacture or have replaceable filters. The results of swab analysis however showed no difference in numbers of colonies between these two podiatrist and other podiatrists. This would indicate that they were still exposed in the airborne fungal spores after they have removed their masks. However, the limited sample size makes it difficult to draw any major conclusions from this sub-group.

## Discussion

It is known that up to 100,000 different fungal species have been identified with many being common indoor and outdoor fungi [14]. The results from the podiatrists and the controls showed many samples containing *Penicillium spp.* and *Aspergillus spp*. which are prevalent in the environment and are encompassed in the hyaline hyphomycetes group of fungi [14,24]. Although prevalent, organisms from this fungi group are responsible for causing both localised and disseminated infections with some species found in infected nails. The podiatrists’ culture results identified 44% as having *Aspergillus fumigatus* in their noses in comparison to only 9% in the control group.

*Aspergillus fumigatus* is a known nail pathogen and is responsible for most cases of invasive, non-invasive and the allergic form of aspergillosis, which due to its small spore (conidia) size of less than 5 μm, which can easily pass through to the lungs of the podiatrist [25]. Both groups had fairly equal amounts of *Penicillium* cultured and a number of other unidentified hyaline hyphomycetes again common in the general environment.

The relatively large number of the dematiaceous fungi found in the subjects was of interest as this group of fungi is made up of many of the common environmental moulds such as *Cladosporium*, *Alternaria*, *Exophiala* and *Phaeoannellomyces werneckii*. Of these, *Scytilidium* and *Alternaria* which were both found in the podiatrists, are known to cause onychomycosis and have been implicated in other skin infections. *Phaeoannellomyces werneckii* is a common saprophytic fungus found in soils and compost that can cause superficial fungal infections of the skin [13] in subjects with immunocompromised health.

The control group had 44% of unidentified yeasts cultured from their nasal swabs compared to only 6% of the podiatrists. Yeasts, like moulds, are common in the environment but in contrast to moulds, are mostly anaerobes. The significance of the difference between these two groups is unclear as yeasts are also known nail contaminants and should therefore be found in the nasal cavities of the podiatrists at the same levels as the control group. Previous studies [15] have reported an immune response (antibodies to the *Tricophyton* antigens) in podiatrists. It is speculated that perhaps this could be the same for the yeasts antigens. Large numbers of yeasts have been found in nail dust and are known opportunistic pathogens especially in compromised hosts [26]. It is however important to consider that all but one of the control group were women, this is compared to 70% of the population of the podiatry group who were female. This may also explain the higher incidence of yeast as women are more predisposed to yeast infections due to the use of oral contraceptives [13].

As identified by earlier studies, bacteria has been found to be in human nail dust [2,16]. These results showed two different types of bacteria cultured from the noses of podiatrists. *Staphylococcus aureus* is known to be carried in many people’s noses [13]. This particular strain showed resistance to penicillin and ampicillin and was found by the commercial laboratory culture. This resistant organism found in the nasal cavity of a podiatrist highlights the need for thorough infection control in the podiatry profession as this represents a significant risk to a health compromised patient attending their clinic.

Of interest was one unusual dermatophyte cultured in this study, *Microsporum ferrugineum* is regarded to be a rare organism [27] and is the cause of tinea capitis, which can be easily transferred between humans [28]. It should be noted that this culture was one of the six sent to an accredited pathology laboratory for a second opinion.

It is not surprising that only one dermatophyte was cultured in this study. Previous studies all reported the nil growth of dermatophytes even using the recommended culture medium as in this study [2,16]. Dermatophytes invade keratinised tissue mostly found on dry non mucosal skin. The nose is lined with mucous membranes with mucous-coated hairs it may be they do not survive for long in that environment [13]. McLarnon [16] found the absence of dermatophyte fungi unusual given that many podiatrists are sensitised to *Trichophyton rubrum* supposedly from infected toenails. Inhalation is the most probable route for sensitisation to occur so it is assumed the spores must be present in the air and therefore in the nose of a podiatrist. It has also been found the elderly who make up a large percentage of podiatric clientele tend to have their nails colonised by saprophytic moulds rather than dermatophytes and so podiatrists may be more likely to encounter moulds instead [2,16].

External validation of this study can be seen by the fact that similar types of fungi were found in this study as compared with the studies undertaken by Purkiss [2] and McLarnon [16] in which *Aspergillus fumigatus* and *Penicillium* were the major organisms cultured with no dermatophytes isolated. A study by Eastman and Handtke [29] found *Penicillium*, *Cladosporium* and *Exophiala* prevalent in their culturing with *Aspergillus versicolor*, rather than *Aspergillus fumigatus*, isolated and in a control subject only. This species of *Aspergillus* was isolated only in the control group of this study. The Eastman study also had a large contingent of students that made up the podiatry group, which due to their lower exposure rate to nail dust could help attribute to the difference. This could also be explained by error in the identification of the fungi in either study as the identification of species of *Aspergillus* can be difficult due to its large genus [25].

When examining the demographics of the podiatry group we see that thirty one of the podiatrists had been practising for under 20 years compared with 18 for over 20 years. The number of times they had used a nail drill in the previous two weeks was quite varied, with eleven out of the fifty podiatrists not having used the drill at all for two weeks. This compared with ten podiatrists who used the drill between eighty to a hundred times per week, this may reflect the nature and type of practice which each podiatrist had. Podiatrists who had been in practice for longer than twenty years would see more older patients with nail pathology compared with younger podiatrists. This observation would be based on a change in the type of podiatry practice over the last ten years with far more biomechanical treatments as part of general practice. However this is only anecdotal and deserves more research. Every drill had dust extraction fitted, except one, that was used for occasional domiciliary care only.

Of the 47 podiatrists who responded to the question regarding the types of masks worn, 23 wore simple disposable with eight podiatrists not wearing a mask at all. Of the 15 podiatrists who drilled more than eighty one times in the previous two weeks, there were only two (14%) who did not wear a mask, this seems completely negligent of their own health and safety and long term practice. There were only three podiatrists who wore more specialised masks than the basic surgical and simple disposable type in the study. These masks were changed less frequently than weekly which is probably standard for that type of respirator. According to Purkiss [2], the surgical and simple disposable masks are inadequate to filter dust particles less than 3 μm and even less inadequate when 'wet’ from normal respiration moisture after a short time frame. The questionnaire result showed that only half of the podiatrists who used these types of masks changed them after each patient. Changing the mask this frequently is adequate for basic level filtering and for cross infection purposes only [2].

Whilst the majority (83%) of podiatrists in this study used a mask whilst operating a nail drill, the efficacy of the type of masks mainly worn may not be adequate to filter the particle size of human nail dust. Those not using masks are seriously compromising their health and need to be counselled into better practice. The problems of further reduced filtration and cross infection with the simple disposable and surgical masks are compounded if they are not changed frequently, ideally after each patient [30]. However, full protective cartridge type high filtration masks maybe the best option along with air cleaning equipment within the podiatry practice [31]. Alternatively completely stopping nail reduction with high speed burrs and using alternative means of reduction maybe the answer to better health in podiatrists.

A limitation of the study may have been the nasal sampling technique used, this may have been compromised due to the individual degrees of comfort/discomfort, all subjects self-administered the swabbing of their noses and although there were instructions on the procedure it was anticipated that some may not have swabbed far enough into the cavity and therefore may have missed microbes. Future research may need to use a single researcher to take all samples form subjects to minimises any perceived data collection errors. The convenience sample size was relatively small and selective and cannot be deemed representative of the normal population of podiatrists as many were rural podiatrists and therefore possibly exposed to greater levels of environmental organisms.

The procedure of culturing and analysing the microbes also posed limitations. As the plates were not inoculated in a sterile room, contaminants may have entered the plates during the inoculation when the lids were lifted, however this is seen as a minor risk and the same potential error for both subject groups.As the collection of data (nasal swabbing) occurred only once, there is a possibility that the time of the year in which the data collection occurred could impact on the types of nasal flora present in the samples. However, this would be apparent in both samples which were tested within one week of each other. The results may be different to another study that is carried out in the winter months. Variations in naturally occurring environmental fungal spores may affect the transferability of the study results due to the different regions the podiatrists and controls resided in as there are some differences in fungal spores occurring within different regions in Australia [32,33].

## Conclusion

This study found that podiatrists when compared to a control group had more types of microbes present in their noses with common and serious pathogen, *Aspergillus fumigatus* found to be more prevalent within the podiatric group*.* The controls did however have more numbers of moulds/fungi cultured but most found have a relative low association with disease at low numbers and in healthy hosts. The finding of a rare dermatophyte that is the cause of tinea capitus, invading the skin and hair rather than the nails also questions the risks of inhaling airborne dust and matter from skin during debriding the skin with high speed sanding disc such as the Moore’s disc which is a common podiatric practice.

## Competing interests

The authors declare that they have no competing interests.

## Authors’ contributions

All authors contributed equally to this publication. All authors read and approved the final manuscript.
